# Convergence rates in the law of large numbers for long-range dependent linear processes

**DOI:** 10.1186/s13660-017-1517-6

**Published:** 2017-10-02

**Authors:** Tao Zhang, Pingyan Chen, Soo Hak Sung

**Affiliations:** 1 Department of Statistics, Jinan University, Guangzhou, 510630 P.R. China; 2 Department of Mathematics, Jinan University, Guangzhou, 510630 P.R. China; 3 Department of Applied Mathematics, Pai Chai University, Daejeon, 35345 South Korea

**Keywords:** 60F15, linear process, convergence rate, Marcinkiewicz-Zygmund law of large numbers

## Abstract

Baum and Katz (Trans. Am. Math. Soc. 120:108-123, 1965) obtained convergence rates in the Marcinkiewicz-Zygmund law of large numbers. Their result has already been extended to the short-range dependent linear processes by many authors. In this paper, we extend the result of Baum and Katz to the long-range dependent linear processes. As a corollary, we obtain convergence rates in the Marcinkiewicz-Zygmund law of large numbers for short-range dependent linear processes.

## Introduction

There are many literature works concerning the convergence rates in the Marcinkiewicz-Zygmund law of large numbers. One can refer to Alf [2], Alsmeyer [3], Baum and Katz [1], Heyde and Rohatgi [4], Hu and Weber [5], Rohatgi [6], and so on.

Baum and Katz [1] obtained the following convergence rates in the Marcinkiewicz-Zygmund law of large numbers.

### Theorem 1.1

Baum and Katz [1]

*Let* $r\geq1$, $1\leq p<2$ *and* $\{X, X_{n}, n\geq1 \}$ *be a sequence of independent and identically distributed* (*i*.*i*.*d*.) *random variables*. *Then* $EX=0$ *and* $E \vert X \vert ^{rp}<\infty$ *imply*

$$\sum^{\infty}_{n=1}n^{r-2}P \Biggl( \Biggl\vert \sum_{k=1}^{n} X_{k} \Biggr\vert >n^{1/p}\varepsilon \Biggr)< \infty \quad\textit{for all } \varepsilon>0. $$

When $r=2$, the cases of $p=1$ and $1\leq p<2$ have already been proved by Hsu and Robbins [7] and Katz [8], respectively.

Let $\{\zeta_{i},i\in\Bbb {Z}\}$ be a sequence of i.i.d. random variables and $\{a_{i},i\in\Bbb {Z}\}$ be a sequence of real numbers. Here and in the following, $\Bbb {Z}$ denotes the set of all integers. Then $\{X_{n}, n\ge1\}$ is called a linear process or an infinite order moving average process if $X_{n}$ is defined by

1.1 $$\begin{aligned} X_{n}=\sum^{\infty}_{i=-\infty}a_{i+n} \zeta_{i} \quad\text{for $n\geq1$.} \end{aligned}$$

If $\sum^{\infty}_{i=-\infty} \vert a_{i} \vert <\infty$, then $\{X_{n}, n\geq1\}$ has short memory or is short-range dependent. If $\sum^{\infty}_{i=-\infty} \vert a_{i} \vert =\infty$, then $\{X_{n}, n\geq1\}$ has long memory or is long-range dependent (see Chapter 3 in Giraitis et al. [9]).

In the short-range dependent case, Koopmans [10] showed that if $\zeta _{0}$ has the moment generating function, then the strong law of large numbers for the linear process holds with exponential convergence rate. Hanson and Koopmans [11] generalized this result to a class of linear processes of independent but non-identically distributed random variables $\{\zeta_{i}, i\in\Bbb {Z}\}$ and to arbitrary subsequences of $\{X_{n}, n\ge1\}$. Li et al. [12] extended Katz [8] theorem to the setting of short-range dependent linear processes.

### Theorem 1.2

Li et al. [12]

*Let* $1\le p<2$. *Let* $\{a_{i}, i \in\Bbb {Z}\}$ *be an absolutely summable sequence of real numbers*. *Suppose that* $\{ X_{n}, n\geq1\}$ *is the linear process of a sequence* $\{ \zeta_{i}, i \in\Bbb {Z}\}$ *of i*.*i*.*d*. *random variables with mean zero and* $E \vert \zeta_{0} \vert ^{2p}<\infty$. *Then*

$$\sum^{\infty}_{n=1} P\Biggl( \Biggl\vert \sum ^{n}_{k=1}X_{k} \Biggr\vert >n^{1/p} \varepsilon\Biggr)< \infty \quad\textit{for all }\varepsilon>0. $$

Note that Theorem 1.2 corresponds to Theorem 1.1 with $r=2$. Zhang [13] extended Theorem 1.1 with $r>1$ to the short-range dependent linear process of a sequence of identically distributed *φ*-mixing random variables. Since independent random variables are also *φ*-mixing, it follows by Zhang [13] theorem that Theorem 1.2 also holds for $r>1$.

In this paper, we obtain convergence rates in the Marcinkiewicz-Zygmund law of large numbers for long-range dependent linear processes of i.i.d. random variables. For convenience of notation, let

$$W_{n}(t)= \Biggl(\sum^{\infty}_{i=-\infty} \vert \omega_{ni} \vert ^{t} \Biggr)^{1/t} \quad\text{for }n\geq1\mbox{ and }t>0, $$

where $\omega_{ni}=\sum^{n}_{k=1}a_{i+k}$. In the long-range dependent case, Characiejus and Račkauskas [14] obtained the convergence rate in the Marcinkiewicz-Zygmund law of large numbers for the linear process $\{ Y_{n}, n\ge1 \}$ which is slightly different from (1.1) and defined by

1.2 $$\begin{aligned} Y_{n}=\sum_{i=0}^{\infty}a_{i} \zeta_{n-i} \quad\text{for $n\geq1$,} \end{aligned}$$

where $a_{i}=0$ if $i<0$.

### Theorem 1.3

Characiejus and Račkauskas [14]

*Let* $\{ Y_{n}, n\geq1 \}$ *be defined as above and* $1< p<2$. *Let* $\{a_{i}, i \in\Bbb {Z}\}$ *be a sequence of real numbers such that*

$$\sum^{\infty}_{i=-\infty} \vert a_{i} \vert ^{p}< \infty, $$

*where* $a_{i}=0$ *if* $i<0$. *Assume that*

$$W_{n}(q)/W_{n}(p)=O\bigl(n^{1/q-1/p}\bigr) \quad\textit{for some }q\in(p,2]. $$

*If* $E\zeta_{0}=0$ *and* $E[ \vert \zeta_{0} \vert ^{p}\log(1+ \vert \zeta_{0} \vert )]<\infty$, *then*

1.3 $$\begin{aligned} \sum^{\infty}_{n=1}n^{-1}P \Biggl( \Biggl\vert \sum^{n}_{k=1}Y_{k} \Biggr\vert >W_{n}(p)\varepsilon \Biggr)< \infty\quad \textit{for all } \varepsilon>0. \end{aligned}$$

The above theorem shows a convergence rate in the Marcinkiewicz-Zygmund weak law of large numbers with the norming sequence $W_{n}(p)$.

We now compare Theorem 1.3 with Theorem 1.1. Since Theorem 1.3 deals with only the case $r=1$, it is interesting to prove that Theorem 1.3 holds for the case $r>1$. When $r=1$, Theorem 1.1 requires a finite *p*th moment condition, but Theorem 1.3 requires more than finite *p*th moment. To apply Theorem 1.3, it is necessary to estimate $W_{n}(p)$. If $\{a_{i}, i \in\Bbb {Z}\}$ is an absolutely summable sequence, then we have, by the result of Burton and Dehling [15] (see also Lemma 2.4), that for any $t>0$

$$\frac{1}{n} W_{n}^{t}(t) \to\sum _{i=-\infty}^{\infty}a_{i}, $$

and hence (1.3) holds with $W_{n}(p)$ replaced by $n^{1/p}$. However, for the long-range dependent case, it is not easy to estimate $W_{n}(t)$.

In this paper, we extend Theorem 1.1 to the long-range dependent linear processes. As a corollary, we obtain a long-range dependent setting of Theorem 1.2. Further, we propose a method to estimate $W_{n}(t)$ for the long-range dependent case.

Throughout this paper, *C* denotes a positive constant which may vary at each occurrence. For events *A* and *B*, $I(A)$ denotes the indicator function of the event *A*, and $I(A,B)=I(A\cap B)$.

## Convergence of long-range dependent linear processes

In this section, we extend Theorem 1.1 to the long-range dependent linear processes. To prove the main results, we need the following lemmas. The first one is the von Bahr-Esseen inequality (see von Bahr and Esseen [16]). The second is known as Fuk-Nagaev inequality (see Corollary 1.8 in Nagaev [17]).

### Lemma 2.1

*Let* $\{\zeta_{i}, i \geq1\}$ *be a sequence of independent random variables with* $E\zeta_{i}=0$ *and* $E \vert \zeta_{i} \vert ^{t}<\infty $ *for some* $1\leq t \leq2$. *Then*, *for all* $n\geq1$,

$$E \Biggl\vert \sum^{n}_{i=1} \zeta_{i} \Biggr\vert ^{t}\leq C_{t}\sum ^{n}_{i=1}E \vert \zeta_{i} \vert ^{t}, $$

*where* $C_{t}>0$ *is a positive constant depending only on* *t*.

### Lemma 2.2

*Let* $\{\zeta_{i}, i\geq1\}$ *be a sequence of independent random variables with* $E\zeta_{i}=0$. *Then*, *for any* $t\ge2$ *and* $x>0$,

$$P \Biggl( \Biggl\vert \sum^{n}_{i=1} \zeta_{i} \Biggr\vert >x \Biggr)\le(1+2/t)^{t} x^{-t}\sum^{n}_{i=1}E \vert \zeta_{i} \vert ^{t}+2\exp \biggl\{ -\frac{2x^{2}}{(t+2)^{2} e^{t} \sum^{n}_{i=1} \operatorname{Var}(\zeta_{i})} \biggr\} . $$

The following lemma is well known and can be easily proved by using a standard method.

### Lemma 2.3

*Let* $p>0$ *and* *ζ* *be a random variable*. *Then the following statements hold*.

(i) *If* $0<\theta<p$, *then* $\sum_{n=1}^{\infty}n^{-\theta/p} E \vert \zeta \vert ^{\theta}I( \vert \zeta \vert >n^{1/p})\le CE \vert \zeta \vert ^{p}$.
(ii) *If* $p< q$, *then* $\sum_{n=1}^{\infty}n^{-q/p} E \vert \zeta \vert ^{q} I( \vert \zeta \vert \le n^{1/p})\le CE \vert \zeta \vert ^{p}$.
(iii) *If* $r>1$, *then* $\sum_{n=1}^{\infty}n^{r-2} E \vert \zeta \vert ^{p}I( \vert \zeta \vert >n^{1/p})\le CE \vert \zeta \vert ^{rp}$.
(iv) *If* $rp< q$, *then* $\sum_{n=1}^{\infty}n^{r-1-q/p} E \vert \zeta \vert ^{q}I( \vert \zeta \vert \le n^{1/p})\le CE \vert \zeta \vert ^{rp}$.

The following lemma is useful to estimate $W_{n}(t)$ when the sequence $\{ a_{i}, i \in\Bbb {Z}\}$ is absolutely summable. However, it is not applicable to the long-range dependent case.

### Lemma 2.4

Burton and Dehling [15]

*Let* $\sum_{i=-\infty }^{\infty}a_{i}$ *be an absolutely convergent series of real numbers with* $a=\sum_{i=-\infty}^{\infty}a_{i}$. *Then*, *for any* $t>0$,

$$\lim_{n\to\infty} \frac{1}{n} \sum _{i=-\infty}^{\infty} \vert \omega_{ni} \vert ^{t}= \vert a \vert ^{t}, $$

*where* $\omega_{ni}=\sum_{k=1}^{n} a_{i+k}$.

We now state and prove our main results. The first theorem treats the case $r>1$.

### Theorem 2.1

*Let* $r>1$ *and* $1\leq p<2$. *Let* $\{a_{i}, i \in\Bbb {Z}\}$ *be a sequence of real numbers with*

$$\sum^{\infty}_{i=-\infty} \vert a_{i} \vert ^{p}< \infty. $$

*Suppose that* $\{ X_{n}, n\geq1\}$ *is the linear process of a sequence* $\{ \zeta_{i}, i \in\Bbb {Z}\}$ *of i*.*i*.*d*. *random variables with mean zero and* $E \vert \zeta_{0} \vert ^{rp}<\infty$. *Furthermore*, *assume that one of the following conditions holds*.

1. *If* $1< rp<2$, *then*
  $$W_{n}(q)/W_{n}(p)=O\bigl(n^{1/q-1/p}\bigr)\quad \textit{for some }q \in(rp, 2). $$
2. *If* $rp\geq2$, *then*
  $$W_{n}(q)/W_{n}(p)=O\bigl(n^{1/q-1/p}\bigr)\quad \textit{for some }q>rp $$
  *and*
  $$W_{n}(s)/W_{n}(p)=o\bigl((\log n)^{-1/s}\bigr)\quad \textit{for some }s\in(p,2]. $$
  *Then*
  $$\sum^{\infty}_{n=1}n^{r-2}P\Biggl( \Biggl\vert \sum^{n}_{k=1}X_{k} \Biggr\vert >W_{n}(p)\varepsilon\Biggr)< \infty \quad\textit{for all } \varepsilon>0. $$

### Proof

(1) For each $n\geq1$, we have

$$\begin{aligned} \sum^{n}_{k=1}X_{k} ={}&\sum ^{\infty}_{i=-\infty}\sum^{n}_{k=1}a_{i+k} \zeta_{i} =\sum^{\infty}_{i=-\infty} \omega_{ni}\zeta_{i} \\ ={}&\sum^{\infty}_{i=-\infty}\omega_{ni} \bigl[\zeta_{i} I\bigl( \vert \zeta_{i} \vert >n^{1/p}\bigr)-E\zeta _{i} I\bigl( \vert \zeta_{i} \vert >n^{1/p}\bigr)\bigr] \\ &{} +\sum^{\infty}_{i=-\infty}\omega_{ni} \bigl[\zeta_{i} I\bigl( \vert \zeta_{i} \vert \leq n^{1/p}\bigr)-E\zeta_{i} I\bigl( \vert \zeta_{i} \vert \leq n^{1/p}\bigr)\bigr] \\ :={}&S_{n}'+S_{n}'' \end{aligned}$$

and hence,

2.1 $$\begin{aligned} &\sum^{\infty}_{n=1}n^{r-2}P\Biggl( \Biggl\vert \sum^{n}_{k=1}X_{k} \Biggr\vert >W_{n}(p)\varepsilon\Biggr) \\ &\quad\leq\sum^{\infty}_{n=1}n^{r-2}P\bigl( \bigl\vert S_{n}' \bigr\vert >W_{n}(p) \varepsilon/2\bigr) +\sum^{\infty}_{n=1}n^{r-2}P \bigl( \bigl\vert S_{n}'' \bigr\vert >W_{n}(p)\varepsilon/2\bigr). \end{aligned}$$

By the Markov inequality, Lemmas 2.1 and 2.3, we have

$$\begin{aligned} \sum^{\infty}_{n=1}n^{r-2}P\bigl( \bigl\vert S_{n}' \bigr\vert >W_{n}(p) \varepsilon/2\bigr) &\leq\sum^{\infty}_{n=1}n^{r-2} \frac{2^{p}E \vert S_{n}' \vert ^{p}}{\varepsilon^{p} W^{p}_{n}(p)} \\ &\leq C\sum^{\infty}_{n=1}n^{r-2} \frac{\sum^{\infty}_{i=-\infty} \vert \omega _{ni} \vert ^{p} E \vert \zeta_{0} \vert ^{p}I( \vert \zeta_{0} \vert >n^{1/p})}{\sum^{\infty}_{i=-\infty } \vert \omega_{ni} \vert ^{p}} \\ &=C\sum^{\infty}_{n=1}n^{r-2}E \vert \zeta_{0} \vert ^{p}I\bigl( \vert \zeta_{0} \vert >n^{1/p}\bigr) \\ &\leq CE \vert \zeta_{0} \vert ^{rp}< \infty. \end{aligned}$$

Thus the first series on the right-hand side of (2.1) converges.

Similarly, by the Markov inequality, Lemmas 2.1 and 2.3, we have

$$\begin{aligned} \sum^{\infty}_{n=1}n^{r-2}P\bigl( \bigl\vert S_{n}'' \bigr\vert >W_{n}(p)\varepsilon/2\bigr) &\leq\sum^{\infty}_{n=1}n^{r-2} \frac{2^{q}E \vert S_{n}'' \vert ^{q}}{\varepsilon^{q} W^{q}_{n}(p)} \\ &\leq C\sum^{\infty}_{n=1}n^{r-2} \frac{\sum^{\infty}_{i=-\infty} \vert \omega _{ni} \vert ^{q} E \vert \zeta_{0} \vert ^{q}I( \vert \zeta_{0} \vert \leq n^{1/p})}{W^{q}_{n}(p)} \\ &=C\sum^{\infty}_{n=1}n^{r-2} \biggl( \frac{W_{n}(q)}{W_{n}(p)} \biggr)^{q} E \vert \zeta _{0} \vert ^{q}I\bigl( \vert \zeta_{0} \vert \leq n^{1/p} \bigr) \\ &\leq C\sum^{\infty}_{n=1}n^{r-2} \bigl(n^{1/q-1/p}\bigr)^{q} E \vert \zeta_{0} \vert ^{q}I\bigl( \vert \zeta _{0} \vert \leq n^{1/p} \bigr) \\ &=C\sum^{\infty}_{n=1}n^{r-1-q/p} E \vert \zeta_{0} \vert ^{q}I\bigl( \vert \zeta_{0} \vert \leq n^{1/p}\bigr) \\ &\leq CE \vert \zeta_{0} \vert ^{rp}< \infty. \end{aligned}$$

Hence the second series on the right-hand side of (2.1) also converges.

(2) For each $n\geq1$, we have

$$\begin{aligned} \sum^{n}_{k=1}X_{k} ={}&\sum ^{\infty}_{i=-\infty}\sum^{n}_{k=1}a_{i+k} \zeta_{i} =\sum^{\infty}_{i=-\infty} \omega_{ni}\zeta_{i} \\ ={}&\sum^{\infty}_{i=-\infty}\bigl[ \omega_{ni}\zeta_{i} I\bigl( \vert \omega_{ni} \zeta _{i} \vert >W_{n}(p)\bigr)-E\omega_{ni} \zeta_{i} I\bigl( \vert \omega_{ni}\zeta_{i} \vert >W_{n}(p)\bigr)\bigr] \\ &{} +\sum^{\infty}_{i=-\infty}\bigl[ \omega_{ni}\zeta_{i} I\bigl( \vert \omega_{ni} \zeta _{i} \vert \leq W_{n}(p)\bigr)-E\omega_{ni} \zeta_{i} I\bigl( \vert \omega_{ni}\zeta_{i} \vert \leq W_{n}(p)\bigr)\bigr] \\ :={}&T_{n}'+T_{n}'' \end{aligned}$$

and hence,

2.2 $$\begin{aligned} &\sum^{\infty}_{n=1}n^{r-2}P\Biggl( \Biggl\vert \sum^{n}_{k=1}X_{k} \Biggr\vert >W_{n}(p)\varepsilon\Biggr) \\ &\quad\leq\sum^{\infty}_{n=1}n^{r-2}P\bigl( \bigl\vert T_{n}' \bigr\vert >W_{n}(p) \varepsilon/2\bigr) +\sum^{\infty}_{n=1}n^{r-2}P \bigl( \bigl\vert T_{n}'' \bigr\vert >W_{n}(p)\varepsilon/2\bigr). \end{aligned}$$

By the Markov inequality, Lemmas 2.1 and 2.3, we have

$$\begin{aligned} &\sum^{\infty}_{n=1}n^{r-2}P\bigl( \bigl\vert T_{n}' \bigr\vert >W_{n}(p) \varepsilon/2\bigr) \\ &\quad\leq\sum^{\infty}_{n=1}n^{r-2} \frac{2^{p}E \vert T_{n}' \vert ^{p}}{\varepsilon^{p} W^{p}_{n}(p)} \\ &\quad\leq C\sum^{\infty}_{n=1}n^{r-2} \frac{\sum^{\infty}_{i=-\infty}E \vert \omega _{ni}\zeta_{i} \vert ^{p} I( \vert \omega_{ni}\zeta_{i} \vert > W_{n}(p))}{W^{p}_{n}(p)} \\ &\quad=C\sum^{\infty}_{n=1}n^{r-2} \frac{\sum^{\infty}_{i=-\infty}E \vert \omega _{ni}\zeta_{i} \vert ^{p} I( \vert \omega_{ni}\zeta_{i} \vert > W_{n}(p), \vert \zeta _{i} \vert >n^{1/p})}{W^{p}_{n}(p)} \\ &\qquad{} +C\sum^{\infty}_{n=1}n^{r-2} \frac{\sum^{\infty}_{i=-\infty}E \vert \omega _{ni}\zeta_{i} \vert ^{p} I( \vert \omega_{ni}\zeta_{i} \vert > W_{n}(p), \vert \zeta_{i} \vert \leq n^{1/p})}{W^{p}_{n}(p)} \\ &\quad\leq C\sum^{\infty}_{n=1}n^{r-2} \frac{\sum^{\infty}_{i=-\infty} \vert \omega _{ni} \vert ^{p}E \vert \zeta_{i} \vert ^{p} I( \vert \zeta_{i} \vert >n^{1/p})}{W^{p}_{n}(p)} \\ &\qquad{}+C\sum^{\infty}_{n=1}n^{r-2} \frac{\sum^{\infty}_{i=-\infty }E[ \vert \omega_{ni}\zeta_{i} \vert ^{p-q} \vert \omega_{ni}\zeta_{i} \vert ^{q} I( \vert \omega_{ni}\zeta _{i} \vert > W_{n}(p), \vert \zeta_{i} \vert \leq n^{1/p})]}{W^{p}_{n}(p)} \\ &\quad\leq C\sum^{\infty}_{n=1}n^{r-2} \frac{\sum^{\infty}_{i=-\infty} \vert \omega _{ni} \vert ^{p}E \vert \zeta_{0} \vert ^{p} I( \vert \zeta_{0} \vert >n^{1/p})}{W^{p}_{n}(p)} \\ &\qquad{}+C\sum^{\infty}_{n=1}n^{r-2} \frac{(W_{n}(p))^{p-q}\sum^{\infty}_{i=-\infty} \vert \omega_{ni} \vert ^{q} E \vert \zeta_{0} \vert ^{q} I( \vert \zeta_{0} \vert \leq n^{1/p})}{W^{p}_{n}(p)} \\ &\quad=C\sum^{\infty}_{n=1}n^{r-2}E \vert \zeta_{0} \vert ^{p} I\bigl( \vert \zeta_{0} \vert >n^{1/p}\bigr) \\ &\qquad{}+C\sum^{\infty}_{n=1}n^{r-2} \biggl( \frac{W_{n}(q)}{W_{n}(p)} \biggr)^{q} E \vert \zeta_{0} \vert ^{q}I\bigl( \vert \zeta_{0} \vert \leq n^{1/p} \bigr) \\ &\quad\leq C\sum^{\infty}_{n=1}n^{r-2}E \vert \zeta_{0} \vert ^{p} I\bigl( \vert \zeta_{0} \vert >n^{1/p}\bigr) +C\sum ^{\infty}_{n=1}n^{r-1-q/p}E \vert \zeta_{0} \vert ^{q}I\bigl( \vert \zeta_{0} \vert \leq n^{1/p}\bigr) \\ &\quad\leq CE \vert \zeta_{0} \vert ^{rp}< \infty. \end{aligned}$$

Thus the first series on the right-hand side of (2.2) converges.

We next prove that the second series on the right-hand side of (2.2) converges. We have by Lemma 2.2 that for $t>2$,

2.3 $$\begin{aligned} &\sum^{\infty}_{n=1}n^{r-2}P\bigl( \bigl\vert T_{n}'' \bigr\vert >W_{n}(p)\varepsilon/2\bigr) \\ &\quad\leq C\sum^{\infty}_{n=1}n^{r-2} \frac{\sum^{\infty}_{i=-\infty}E \vert \omega _{ni}\zeta_{i} \vert ^{t} I( \vert \omega_{ni}\zeta_{i} \vert \leq W_{n}(p))}{W^{t}_{n}(p)} \\ & \qquad{}+C\sum^{\infty}_{n=1}n^{r-2}\exp \biggl\{ -\frac{\varepsilon^{2} W_{n}^{2}(p)}{2(t+2)^{2} e^{t} \sum_{i=-\infty}^{\infty}\operatorname{Var}( \omega_{ni}\zeta _{i}I( \vert \omega_{ni}\zeta_{i} \vert \le W_{n}(p)))} \biggr\} . \end{aligned}$$

Hence it is enough to show that two series on the right-hand side of (2.3) converge.

If we take $t>q$, then we have by Lemma 2.3 that

$$\begin{aligned} &\sum^{\infty}_{n=1}n^{r-2} \frac{\sum^{\infty}_{i=-\infty}E \vert \omega_{ni}\zeta _{i} \vert ^{t} I( \vert \omega_{ni}\zeta_{i} \vert \leq W_{n}(p))}{W^{t}_{n}(p)} \\ &\quad=\sum^{\infty}_{n=1}n^{r-2} \frac{\sum^{\infty}_{i=-\infty}E \vert \omega _{ni}\zeta_{i} \vert ^{t} I( \vert \omega_{ni}\zeta_{i} \vert \leq W_{n}(p), \vert \zeta _{i} \vert >n^{1/p})}{W^{t}_{n}(p)} \\ &\qquad{} +\sum^{\infty}_{n=1}n^{r-2} \frac{\sum^{\infty}_{i=-\infty}E \vert \omega _{ni}\zeta_{i} \vert ^{t} I( \vert \omega_{ni}\zeta_{i} \vert \leq W_{n}(p), \vert \zeta_{i} \vert \leq n^{1/p})}{W^{t}_{n}(p)} \\ &\quad=\sum^{\infty}_{n=1}n^{r-2} \frac{\sum^{\infty}_{i=-\infty}E[ \vert \omega _{ni}\zeta_{i} \vert ^{t-p} \vert \omega_{ni}\zeta_{i} \vert ^{p} I( \vert \omega_{ni}\zeta_{i} \vert \leq W_{n}(p), \vert \zeta_{i} \vert >n^{1/p})]}{W^{t}_{n}(p)} \\ & \qquad{}+\sum^{\infty}_{n=1}n^{r-2} \frac{\sum^{\infty}_{i=-\infty}E[ \vert \omega _{ni}\zeta_{i} \vert ^{t-q} \vert \omega_{ni}\zeta_{i} \vert ^{q} I( \vert \omega_{ni}\zeta_{i} \vert \leq W_{n}(p), \vert \zeta_{i} \vert \leq n^{1/p})]}{W^{t}_{n}(p)} \\ &\quad\leq\sum^{\infty}_{n=1}n^{r-2} \frac{(W_{n}(p))^{t-p}\sum^{\infty}_{i=-\infty } \vert \omega_{ni} \vert ^{p}E \vert \zeta_{0} \vert ^{p} I( \vert \zeta_{0} \vert >n^{1/p})}{W^{t}_{n}(p)} \\ &\qquad{} +\sum^{\infty}_{n=1}n^{r-2} \frac{(W_{n}(p))^{t-q}\sum^{\infty}_{i=-\infty} \vert \omega_{ni} \vert ^{q}E \vert \zeta_{0} \vert ^{q} I( \vert \zeta_{0} \vert \leq n^{1/p})}{W^{t}_{n}(p)} \\ &\quad=\sum^{\infty}_{n=1}n^{r-2}E \vert \zeta_{0} \vert ^{p} I\bigl( \vert \zeta_{0} \vert >n^{1/p}\bigr) +\sum^{\infty}_{n=1}n^{r-2} \biggl(\frac{W_{n}(q)}{W_{n}(p)} \biggr)^{q} E \vert \zeta _{0} \vert ^{q}I\bigl( \vert \zeta_{0} \vert \leq n^{1/p}\bigr) \\ &\quad\leq\sum^{\infty}_{n=1}n^{r-2}E \vert \zeta_{0} \vert ^{p} I\bigl( \vert \zeta_{0} \vert >n^{1/p}\bigr) +\sum ^{\infty}_{n=1}n^{r-1-q/p} E \vert \zeta_{0} \vert ^{q}I\bigl( \vert \zeta_{0} \vert \leq n^{1/p}\bigr) \\ &\quad\leq C E \vert \zeta_{0} \vert ^{rp}< \infty. \end{aligned}$$

Hence the first series on the right-hand side of (2.3) converges.

Finally, we show that the second series on the right-hand side of (2.3) converges. Since $p< s\leq2$, we have that

$$\begin{aligned} &\frac{\sum^{\infty}_{i=-\infty} \operatorname{Var}( \omega_{ni}\zeta_{i}I( \vert \omega _{ni}\zeta_{i} \vert \le W_{n}(p)))}{W^{2}_{n}(p)} \\ &\quad\leq\frac{\sum^{\infty}_{i=-\infty} E \vert \omega_{ni}\zeta_{i} \vert ^{2}I( \vert \omega _{ni}\zeta_{i} \vert \le W_{n}(p))}{W^{2}_{n}(p)} \\ &\quad=\frac{\sum^{\infty}_{i=-\infty}E \vert \omega_{ni}\zeta_{i} \vert ^{s+2-s}I( \vert \omega _{ni}\zeta_{i} \vert \leq W_{n}(p))}{W^{2}_{n}(p)} \\ &\quad\leq\frac{(W_{n}(p))^{2-s}\sum^{\infty}_{i=-\infty}E \vert \omega_{ni}\zeta _{i} \vert ^{s}}{W^{2}_{n}(p)} \\ &\quad=\frac{\sum^{\infty}_{i=-\infty} \vert \omega_{ni} \vert ^{s}E \vert \zeta_{0} \vert ^{s}}{W^{s}_{n}(p)} \\ &\quad= \biggl(\frac{W_{n}(s)}{W_{n}(p)} \biggr)^{s}E \vert \zeta_{0} \vert ^{s} \\ &\quad=o(1/\log n), \end{aligned}$$

which implies that

$$\begin{aligned} &\sum^{\infty}_{n=1}n^{r-2} \biggl\{ - \frac{\varepsilon^{2} W_{n}^{2}(p)}{2(t+2)^{2} e^{t} \sum_{i=-\infty}^{\infty}\operatorname{Var}( \omega_{ni}\zeta_{i}I( \vert \omega_{ni}\zeta _{i} \vert \le W_{n}(p)))} \biggr\} \\ &\quad \le C \sum^{\infty}_{n=1}n^{r-2} \biggl\{ -\frac{\varepsilon^{2} \log n}{2(t+2)^{2} e^{t} o(1)} \biggr\} < \infty. \end{aligned}$$

 □

The next theorem treats the case $r=1$.

### Theorem 2.2

*Let* $1\leq p<2$. *Let* $\{a_{i}, i \in\Bbb {Z}\}$ *be a sequence of real numbers with*

$$\sum^{\infty}_{i=-\infty} \vert a_{i} \vert ^{\theta}< \infty\quad\textit{for some }0< \theta< p. $$

*Suppose that* $\{ X_{n}, n\geq1\}$ *is the linear process of a sequence* $\{ \zeta_{i}, i \in\Bbb {Z}\}$ *of i*.*i*.*d*. *random variables with mean zero and* $E \vert \zeta_{0} \vert ^{p}<\infty$. *Furthermore*, *assume that*

$$W_{n}(\theta)/W_{n}(p)=O\bigl(n^{1/\theta-1/p}\bigr) $$

*and*

$$W_{n}(q)/W_{n}(p)=O\bigl(n^{1/q-1/p}\bigr)\quad\textit{for some }q \in(p, 2). $$

*Then*

$$\sum^{\infty}_{n=1}n^{-1}P\Biggl( \Biggl\vert \sum^{n}_{k=1}X_{k} \Biggr\vert >W_{n}(p)\varepsilon\Biggr)< \infty \quad\textit{for all } \varepsilon>0. $$

### Proof

The proof is similar to that of Theorem 2.1(1). We proceed with two cases $1\leq\theta< p$ and $0<\theta<1$.

For the case $1\leq\theta< p$, we have by Lemmas 2.1 and 2.3 that

$$\begin{aligned} \sum^{\infty}_{n=1}n^{-1}P\bigl( \bigl\vert S_{n}' \bigr\vert >W_{n}(p) \varepsilon/2\bigr) &\leq\sum^{\infty}_{n=1}n^{-1} \frac{2^{\theta}E \vert S_{n}' \vert ^{\theta}}{\varepsilon ^{\theta}W^{\theta}_{n}(p)} \\ &\leq C\sum^{\infty}_{n=1}n^{-1} \frac{\sum^{\infty}_{i=-\infty} \vert \omega _{ni} \vert ^{\theta}E \vert \zeta_{0} \vert ^{\theta}I( \vert \zeta_{0} \vert >n^{1/p})}{W^{\theta}_{n}(p)} \\ &=C\sum^{\infty}_{n=1}n^{-1} \biggl( \frac{W_{n}(\theta)}{W_{n}(p)} \biggr)^{\theta}E \vert \zeta_{0} \vert ^{p}I\bigl( \vert \zeta_{0} \vert >n^{1/p} \bigr) \\ &\leq C\sum^{\infty}_{n=1}n^{-1} n^{(1/\theta-1/p)\theta} E \vert \zeta _{0} \vert ^{p}I\bigl( \vert \zeta_{0} \vert >n^{1/p}\bigr) \\ &\leq CE \vert \zeta_{0} \vert ^{p}< \infty. \end{aligned}$$

As in the proof of Theorem 2.1(1), we have that

$$\sum^{\infty}_{n=1}n^{-1}P\bigl( \bigl\vert S_{n}'' \bigr\vert >W_{n}(p)\varepsilon/2\bigr)\le C E \vert \zeta _{0} \vert ^{p}< \infty. $$

For the case $0<\theta<1$, we rewrite $\sum_{k=1}^{n} X_{k}$ as

$$\begin{aligned} \sum_{k=1}^{n} X_{k} ={}&\sum ^{\infty}_{i=-\infty}\omega_{ni} \zeta_{i} I\bigl( \vert \zeta_{i} \vert >n^{1/p} \bigr)+\sum^{\infty}_{i=-\infty}\omega_{ni} \bigl[\zeta_{i} I\bigl( \vert \zeta_{i} \vert \leq n^{1/p}\bigr)-E\zeta _{i} I\bigl( \vert \zeta_{i} \vert \leq n^{1/p}\bigr)\bigr] \\ & {}-\sum^{\infty}_{i=-\infty}\omega_{ni} E \zeta_{i} I\bigl( \vert \zeta _{i} \vert >n^{1/p} \bigr) \\ :={}&S_{n}'+S_{n}''-S_{n}'''. \end{aligned}$$

If $0<\theta<1$, then $\sum_{n=1}^{\infty} \vert a_{n} \vert \leq(\sum_{n=1}^{\infty} \vert a_{n} \vert ^{\theta})^{1/\theta}<\infty$. It follows by Lemma 2.4 that

$$\begin{aligned} W_{n}^{-1}(p) \bigl\vert S_{n}''' \bigr\vert &\leq W_{n}^{-1}(p) \sum ^{\infty}_{i=-\infty} \vert \omega_{ni} \vert E \vert \zeta_{0} \vert I\bigl( \vert \zeta_{0} \vert >n^{1/p}\bigr) \\ &\leq C n^{1-1/p} E \vert \zeta_{0} \vert I\bigl( \vert \zeta_{0} \vert >n^{1/p}\bigr) \\ &\leq C E \vert \zeta_{0} \vert ^{p} I\bigl( \vert \zeta_{0} \vert >n^{1/p}\bigr)\to0 \end{aligned}$$

as $n\to\infty$. Hence

$$\begin{aligned} &\sum^{\infty}_{n=1}n^{-1}P\Biggl( \Biggl\vert \sum^{n}_{k=1}X_{k} \Biggr\vert >W_{n}(p)\varepsilon\Biggr) \\ &\quad \leq C \sum^{\infty}_{n=1}n^{-1}P \bigl( \bigl\vert S_{n}' \bigr\vert >W_{n}(p)\varepsilon/3\bigr) +C\sum^{\infty}_{n=1}n^{-1}P \bigl( \bigl\vert S_{n}'' \bigr\vert >W_{n}(p)\varepsilon/3\bigr). \end{aligned}$$

The rest of the proof is the same as that of the previous case and is omitted. □

The following corollary extends Theorem 1.1 to the short-range dependent linear processes.

### Corollary 2.1

*Let* $r\ge1$, $1\le p<2$, *and* $rp>1$. *Let* $\{a_{i}, i \in\Bbb {Z}\}$ *be an absolutely summable sequence of real numbers*. *Suppose that* $\{ X_{n}, n\geq1\}$ *is the linear process of a sequence* $\{ \zeta_{i}, i \in\Bbb {Z}\}$ *of i*.*i*.*d*. *random variables with mean zero and* $E \vert \zeta_{0} \vert ^{rp}<\infty$. *Then*

$$\sum^{\infty}_{n=1} n^{r-2} P\Biggl( \Biggl\vert \sum^{n}_{k=1}X_{k} \Biggr\vert >n^{1/p} \varepsilon \Biggr)< \infty \quad \textit{for all } \varepsilon>0. $$

### Proof

We first note that

$$\sum_{i=-\infty}^{\infty} \vert a_{i} \vert ^{p}\le \Biggl(\sum_{i=-\infty}^{\infty} \vert a_{i} \vert \Biggr)^{p}< \infty. $$

If $1< p<2$, then we take *θ* such that $1\leq\theta< p$. Then

$$\sum_{i=-\infty}^{\infty} \vert a_{i} \vert ^{\theta}\le \Biggl(\sum_{i=-\infty}^{\infty} \vert a_{i} \vert \Biggr)^{\theta}< \infty. $$

By Lemma 2.4, for any $t>0$, there exist positive constants $C_{1}$ and $C_{2}$ independent of *n* such that

$$C_{1} n^{1/t} \le W_{n}(t) \le C_{2} n^{1/t} \quad\text{for all $n\ge1$.} $$

Then all conditions on $W_{n}(\cdot)$ in Theorems 2.1 and 2.2 are easily satisfied. Hence the proof follows from Theorems 2.1 and 2.2. □

### Remark 2.1

In Corollary 2.1, the case $rp=1$ (i.e., $r=1$ and $p=1$) is not considered. In fact, Corollary 2.1 does not hold for this case (see Sung [18]).

## An estimation of $W_{n}(t)$ for the long-range dependent case

As we have seen in Sections 1 and 2, it is easy to estimate $W_{n}(t)$ for the short-range dependent case. In this section, we propose a method to estimate $W_{n}(t)$ for the long-range dependent case. It is not easy to estimate $W_{n}(t)$ when the sequence $\{a_{i}, i \in \Bbb {Z}\}$ is not absolutely summable. For simplicity, we will consider non-increasing sequences of positive numbers. For the finiteness of $W_{n}(t)$, without loss of generality, it is necessary to assume that $a_{i}=0$ if $i\le0$ and $\sum^{\infty}_{i=1} a_{i}^{t}<\infty$.

### Lemma 3.1

*Let* $t>0$. *Let* $\{a_{i}, i \in\Bbb {Z} \}$ *be a non*-*increasing sequence of positive real numbers satisfying* $a_{i}=0$ *if* $i\leq0$ *and* $\sum^{\infty}_{i=1} a_{i}^{t}<\infty$. *Then*

$$\frac{n}{2} (a_{1}+\cdots+a_{[n/2]} )^{t}+n^{t}\sum_{i=n}^{\infty}a_{i}^{t} \le W_{n}^{t}(t)\le2n (a_{1} +\cdots+ a_{n} )^{t} +n^{t}\sum _{i=n}^{\infty}a_{i}^{t}. $$

### Proof

Since $a_{i}=0$ if $i\le0$ and $0< a_{i}\downarrow$, we get that

$$\begin{aligned} W_{n}^{t}(t) &=\sum_{i=1}^{n} \Biggl(\sum_{j=1}^{i} a_{j} \Biggr)^{t}+ \sum_{i=1}^{n} \Biggl( \sum_{j=1}^{n} a_{i+j} \Biggr)^{t}+\sum_{i=n+1}^{\infty}\Biggl( \sum_{j=1}^{n} a_{i+j} \Biggr)^{t} \\ &\leq2n (a_{1} +\cdots +a_{n} )^{t}+n^{t} \sum_{i=n+1}^{\infty}a_{i+1}^{t} \\ &\leq2n (a_{1} +\cdots +a_{n} )^{t}+n^{t} \sum_{i=n}^{\infty}a_{i}^{t}. \end{aligned}$$

Similarly,

$$\begin{aligned} W_{n}^{t}(t) &=\sum_{i=1}^{n-1} \Biggl(\sum_{j=1}^{i} a_{j} \Biggr)^{t}+ \sum_{i=1}^{\infty}\Biggl( \sum_{j=0}^{n-1} a_{i+j} \Biggr)^{t} \\ &\geq\sum_{i=[n/2]}^{n-1} \Biggl(\sum _{j=1}^{i} a_{j} \Biggr)^{t}+ n^{t}\sum_{i=n}^{\infty}a_{i}^{t} \\ &\geq\frac{n}{2} (a_{1}+\cdots+a_{[n/2]} )^{t}+n^{t}\sum_{i=n}^{\infty}a_{i}^{t}. \end{aligned}$$

Thus the proof is completed. □

The following lemma can be found in Martikainen [19].

### Lemma 3.2

Martikainen [19]

*Let* $\{b_{n}, n\geq1\}$ *be a non*-*decreasing sequence of positive real numbers*. *Then*

$$\sum_{i=n}^{\infty}\frac{1}{ib_{i}}=O \bigl(b_{n}^{-1}\bigr)\quad \Longleftrightarrow \quad\liminf _{n\to\infty} \frac{b_{rn}}{b_{n}}>1\quad \textit{for some integer }r\ge2. $$

Similarly, we can obtain a counterpart of Lemma 3.2.

### Lemma 3.3

*Let* $\{b_{n}, n\geq1\}$ *be a non*-*decreasing sequence of positive real numbers*. *Then*

$$\sum_{i=1}^{n} \frac{b_{i}}{i}=O(b_{n})\quad \Longleftrightarrow\quad \liminf_{n\to \infty} \frac{b_{rn}}{b_{n}}>1 \quad\textit{for some integer }r\ge2. $$

### Proof

The proof is similar to that of Lemma 3.2 and is omitted. □

Using Lemmas 3.2 and 3.3, we have the following lemma.

### Lemma 3.4

*Let* $t>1$ *and let* $\{a_{n}, n\geq1\}$ *be a sequence of positive real numbers satisfying* $na_{n}\uparrow$, $na_{n}^{t}\downarrow$, *and*

$$\frac{1}{r}< \liminf_{n\to\infty} \frac{a_{rn}}{a_{n}}\leq\limsup _{n\to \infty} \frac{a_{rn}}{a_{n}}< \biggl( \frac{1}{r} \biggr)^{1/t} \quad\textit{for some integer }r\ge2. $$

*Then the following statements hold*:

(i) $\sum_{i=n}^{\infty}a_{i}^{t} =O(na_{n}^{t})$.
(ii) $\sum_{i=1}^{n} a_{i} =O(na_{n})$.

### Proof

The proof of (i) follows from Lemma 3.2. The proof of (ii) follows from Lemma 3.3. □

Now we present a method to estimate $W_{n}(t)$ for the long-range dependent case.

### Theorem 3.1

*Let* $t>1$, *and let* $\{a_{n}, n\geq1\}$ *be a sequence of positive real numbers satisfying the same conditions as in Lemma* 3.4. *Then there exist positive constants* $C_{1}$ *and* $C_{2}$ *independent of* *n* *such that*

$$C_{1} n^{1+t} a_{n}^{t}\leq W_{n}^{t}(t)\leq C_{2} n^{1+t} a_{n}^{t} \quad\textit{for all }n\geq1, $$

*where* $a_{i}=0$ *if* $i\le0$.

### Proof

By the condition $na_{n}^{t}\downarrow$, we have $(a_{n+1}/a_{n})^{t}\leq n/(n+1)$, which implies $0< a_{n}\downarrow$. The upper bound of $W_{n}^{t}(t)$ follows by Lemmas 3.1 and 3.4. For the lower bound, we have by $\liminf_{n\to\infty} a_{rn}/a_{n}>1/r$ that

$$a_{rn}/a_{n}\ge1/r \quad \text{for all large $n$.} $$

It follows that for all large *n*

$$n^{t}\sum_{i=n}^{\infty}a_{i}^{t}\geq n^{t}\sum _{i=n}^{rn} a_{i}^{t}\geq(r-1) n^{1+t} a_{rn}^{t}\geq(r-1) r^{-t} n^{1+t}a_{n}^{t}. $$

Since $0< a_{n}\downarrow$,

$$n (a_{1}+\cdots+a_{[n/2]} )^{t} \geq n [n/2]^{t}a_{[n/2]}^{t}\geq n [n/2]^{t}a_{n}^{t}. $$

Hence the lower bound follows from Lemma 3.1. □

Finally, we give two long-range dependent linear processes.

### Example 3.1

Let $a_{i}=1/i$ if $i\geq1$ and $a_{i}=0$ if $i\leq0$. Then the series $\sum_{i=-\infty}^{\infty}a_{i}$ diverges, but $\sum_{i=-\infty}^{\infty}a_{i}^{t}$ converges if $t>1$. Observe that

$$\ln(n+1) \leq\sum_{i=1}^{n} a_{i} \le1+ \ln n. $$

If $t>1$, then

$$\frac{1}{t-1}n^{-t+1} \leq\sum_{i=n}^{\infty}a_{i}^{t}\leq n^{-t}+ \frac {1}{t-1} n^{-t+1}. $$

By Lemma 3.1, for any $t>1$, there exist positive constants $C_{1}$ and $C_{2}$ independent of *n* such that

$$C_{1} n(\ln n)^{t} \leq W_{n}^{t}(t) \leq C_{2} n (\ln n)^{t} \quad\text{for all }n\ge2. $$

Let $X_{n}=\sum_{i=-\infty}^{\infty}a_{i+n}\zeta_{i}$ be the long-range dependent linear process of a sequence $\{\zeta_{i}\}$ of i.i.d. random variables with mean zero and $E \vert \zeta_{0} \vert ^{rp}<\infty$, where $r>1$ and $1< p<2$. Then all conditions of Theorem 2.1 are easily satisfied. By Theorem 2.1,

$$\sum^{\infty}_{n=1}n^{r-2}P\Biggl( \Biggl\vert \sum^{n}_{k=1}X_{k} \Biggr\vert > n^{1/p} \ln n \varepsilon \Biggr)< \infty \quad \text{for all }\varepsilon>0. $$

### Example 3.2

Let $1< p<2$. Let $a_{i}=1/i^{d}$ if $i\geq1$ and $a_{i}=0$ if $i\leq0$, where $1/p< d<1$. Then the series $\sum_{i=-\infty}^{\infty}a_{i}$ diverges, but $\sum_{i=-\infty}^{\infty}a_{i}^{t}$ converges if $t>1/d$. Since $a_{2n}/a_{n} =2^{-d}$, we have by Theorem 3.1 that

$$C_{1} n^{1+t-dt} \leq W_{n}^{t}(t)\leq C_{2} n^{1+t-dt}\quad \text{for all $n\ge1$.} $$

Let $X_{n}=\sum_{i=-\infty}^{\infty}a_{i+n}\zeta_{i}$ be the long-range dependent linear process of a sequence $\{\zeta_{i}\}$ of i.i.d. random variables with mean zero and $E \vert \zeta_{0} \vert ^{p}<\infty$. Take *θ* such that $1/d<\theta<p$. Then all conditions of Theorem 2.2 are easily satisfied. By Theorem 2.2,

$$\sum^{\infty}_{n=1}n^{-1}P\Biggl( \Biggl\vert \sum^{n}_{k=1}X_{k} \Biggr\vert > n^{1/p +1-d} \varepsilon \Biggr)< \infty\quad \text{for all } \varepsilon>0. $$
